# Assessing cancer risk in firefighters in Northern Germany: a retrospective cohort study

**DOI:** 10.1186/s12889-025-24342-3

**Published:** 2025-09-01

**Authors:** Vanessa Sophie Ernst, Kirsi Manz, Kerstin Weitmann, Wolfgang Hoffmann

**Affiliations:** 1 Institute for Community Medicine, Section Epidemiology of Health Care and Community Health, University Medicine Greifswald, Greifswald, Germany; 2 Cancer Registry Mecklenburg-Western Pomerania, Institute for Community Medicine, University Medicine Greifswald, Greifswald, Germany

**Keywords:** Occupational cohort, Firefighters, Cancer registry, Standardized incidence ratio, Standardized mortality ratio

## Abstract

**Background:**

The aim of this study is the analysis of cancer incidence and mortality among the firefighters of the municipal fire brigade in the city of Neubrandenburg in Germany.

**Methods:**

To asses cancer risk standardized incidence ratios and standardized mortality ratios were computed. The risk of the firefighter cohort was compared to the risk of two reference populations: the county Mecklenburg Lake District, where the city of Neubrandenburg is located, and the federal state Mecklenburg-Western Pomerania.

**Results:**

Results are based on data from 100 firefighters. No statistically significant results were observed for the incidence or mortality of all cancers (ICD-10 C00-C97 without C44). The SIR and SMR for bladder cancer were significantly elevated.

**Conclusion:**

The overall cancer incidence and mortality among Neubrandenburg’s firefighters were not higher than among the reference population. The increased incidence and mortality from bladder cancer are in line with published results, especially with the monograph by the International Agency for Research on Cancer on occupational exposure as a firefighter.

## Introduction

Firefighters are exposed to a variety of potential hazards in emergency situations. In addition to fires and explosions, operations also include technical assistance, e.g. for road accidents, operations involving animals as well as rescue and ambulance operations. As many operations involve different exposures, the personal protection of firefighters is of great importance. For years there have been reports of firefighters suffering from cancer, allegedly as a result of their occupational exposure.

In 2010, the WHO’s International Agency for Research on Cancer (IARC) published a detailed list of carcinogenic substances that can be emitted by fires. The IARC emphasizes that all fires produce toxic substances, including known and suspected carcinogens. In addition, soot and dust particles in the smoke act as a transport medium for these substances. The most common substances include benzene, 1,3-butadiene and formaldehyde, but also heavy metals and polycyclic aromatic hydrocarbons (PAHs) [1, 2]. The exposure of firefighters in emergency situations can vary broadly depending on the individual tasks, the type and the duration of an operation. Toxic and carcinogenic substances are mainly absorbed via the respiratory tract. However, absorption via the skin can also be an important route of exposure that needs to be considered [1, 2].

In addition to the analysis of individual exposures and possible routes of exposure, there is a large number of studies and meta-analyses that analyse the incidence of and mortality from cancer in various populations of firefighters. In most of these studies, the incidence or mortality rates of firefighters are compared with the corresponding rates of the general population standardized by age and gender.

For all cancer types combined, a significantly higher incidence was found in female Florida firefighters compared to the overall female Florida population [3]. Analysis of correlations for firefighters in Australia and employees of a fire department training facility in the Australian state of Victoria found a higher incidence of cancer for both groups compared to the general population [4, 5]. For New York firefighters similar results could be found [6]. Firefighters from San Francisco, Chicago and Indiana were found to have a significantly higher mortality rate from all cancers compared to the general population and firefighters in Philadelphia compared to white US-American men [7–9]. This is in contrast to a significantly lower incidence and mortality of cancer among firefighters in Stockholm [10] and in Scotland [11]. Significantly lower mortality rates were also found for Scottish [11], South Korean [12] and Australian [5] firefighters as well as in firefighters who were exposed during the rescue/recovery work at the world trade centre on and after 11 September 2001 [13] compared to the respective reference populations.

In addition to the incidence of and mortality from all cancers, studies also looked at specific types of cancer. For example, a large number of studies found evidence of an increased incidence of bladder cancer among firefighters from Australia [5], Florida [3] and South Korea [14]. This association was supported in two meta-analyses [15, 16]. There is also evidence of increased mortality from bladder cancer among firefighters from Florida [17] and Massachusetts [18] also confirmed in a meta-analysis [16]. There are references in the literature showing significantly higher incidence and mortality rates of cancer of the colon, small intestine and rectum among firefighters compared to the general population or suitable reference groups [7, 8, 14, 18] as well as meta-analyses [15, 16]. Furthermore, the reviewed studies indicate an increased incidence and mortality from cancer of the male reproductive organs [5], testis [3, 4, 15, 19], and prostate [5, 15, 19–22] among firefighters.

The aim of this study is the analysis of cancer incidence and mortality among the firefighters of the municipal fire brigade in the city of Neubrandenburg in Germany, a town with approximately 64,000 inhabitants. The study was initiated by the Ministry of Economy, Labour and Health Mecklenburg-Western Pomerania (MWP) after concerns regarding a high cancer prevalence were voiced by the firefighters themselves, picked up by local media, and brought to the attention of the local government around 2019 [23].

## Methods

### Population

For this retrospective cohort study, we aimed to include all firefighters who worked for the Neubrandenburg municipal fire brigade from January 1, 1990 to December 31, 2016. For the study we developed a detailed data assessment and analysis concept in accordance with the European general data protection regulation (GDPR). This concept was positively reviewed by the ethics committee of the University Medicine Greifswald (UMG, reference number BB 112/21). The name, address of last known residence, date of birth, and period of employment of firefighters during the observation period were recorded on site by an employee of the Human Resource (HR) Department of the city of Neubrandenburg from (archived) personnel files and the required personal data were transfer to the Trusted Third Party (TTP) of the UMG [24] to prepare for requests for their current vital status and an update of their residential address. The currently employed firefighters were informed about the study and their right to actively object to the processing of their data in three information events (one event for each of the three shift groups) carried out by the research team. It was also possible to ask further questions and voice concerns during these three sessions. Subsequently, both the currently employed and the former firefighters received a letter with information on data protection as well as the contact details of the responsible project staff and the data protection officer at UMG. As specified in the study concept the letter stated the opportunity to object.

Only the currently active firefighters, and unknown by us, received a second letter from the employee in the HR department, after she was instructed by the city’s internal legal department to obtain active informed consent for data transfer for the currently employed firefighters.

The identifying information was then transferred to the TTP of the Cancer Registry of Mecklenburg Western Pomerania (CR-MWP). The CR-MWP includes all primary cancers and is thought to be complete. The TTP matched the cohort of firefighters to the CR-MWP database to identify all cancer cases among former and current firefighters. Once this process was completed, a pseudonymised dataset containing the pseudonym, date of birth, date of start and end of employment, date of cancer diagnosis, ICD-10-code of cancer diagnosis, and date of death was provided for the research team for analysis.

The general population of the district of Mecklenburg Lake District (MLD) and the state of MWP were used as references for the analyses. MLD represents a more local reference area while MWP is a broader and more stable reference group. This allows to evaluate potential systematic regional differences. The statistics for the population were provided by the Statistical Office of MWP. A categorisation by gender and 5-year groups was provided for each calendar year of the observation period (1990–2016).

The cause of death statistic was also made available by the Statistical Office for the entire observation period from 1990 to 2016. For the period from 1990 to 1997 the causes of death in the population were available in the ICD coding of revision 9. Since 1998, the statistics have been coded with the ICD coding of revision 10 [25].

### Statistical analysis

The incidence of cancer, all-cause mortality and mortality from cancer in the firefighter cohort were calculated as the standardized incidence ratio (SIR) and standardized mortality ratio (SMR), respectively. Both measures describe the ratio of observed to expected numbers of cases/deaths. Confidence intervals are reported to transparently show the statistical significance/reliability.

The person-years at risk for cancer and/or death were calculated as follows: For the incidence of all cancers, employees were observed from the start of their employment at the fire brigade until they either developed cancer, died without cancer, or reached the end of the observation period without any of these events. For the incidence of cancers in each diagnosis group, employees were observed from the start of their employment at the fire brigade until they either developed a cancer from the respective diagnosis group, died without a cancer from this diagnosis group, or had reached the end of the observation period without any of these events. For example, an individual was considered to be at risk for bladder cancer until that individual was diagnosed with cancer of this organ, died or reached the end of the observation period.

To determine mortality, employees were observed from the start of their employment at the fire department until death or the end of the observation period on December 31, 2016. Employees who could not be definitely identified in the vital status enquiry were censored on the last day of employment at the fire brigade which was used as the last day they were known to be alive.

The diagnosis groups in the SIR and SMR analyses are considered both collectively as the diagnosis category “all cancers (ICD-10 C00-C97 without C44)” and in the individual categories “Digestive organs and peritoneum (gastrointestinal tract) (C15-C26)”, “Liver (C22)”, “Pancreas (C25)”, “Trachea, bronchi and lung (C33-C34)”, “Prostate (C61)”, “Testicles (C62)”, “Bladder (C67)” and “Lymphomas and leukaemia (C81-C96)”. These entities were selected based on significant differences in incidence and mortality between firefighters and the respective reference groups described in the literature. While these entities are mentioned in the literature, it is inconsistent or at times ambiguous whether studies included only C-diagnoses or both C- and D-diagnoses (C-diagnoses refer to invasive tumours, while D-diagnoses refer to in situ tumours). This can be seen in a recent meta-analysis, where a majority of included studies did not specify whether they included in situ diagnoses [26]. Other malignant neoplasms of skin (C44) are excluded from “all cancers” in our study because they are not systematically documented in the cancer registries in Germany. The results only show a cancer entity if there were cases recorded.

## Results

Following the information letter only in one case, relatives of a deceased firefighter objected to the use of his data. This former firefighter was excluded from further analyses.

Following the second letter by the HR department 30 of the active firefighters did not provide consent for their data to be processed. These could therefore not be included in the vital status assessment and further analysis. The TTP received a data set with 101 current and former firefighters from the cohort. As mentioned above one former firefighter was excluded from this data set after obtaining the objection.

**Fig. 1:**
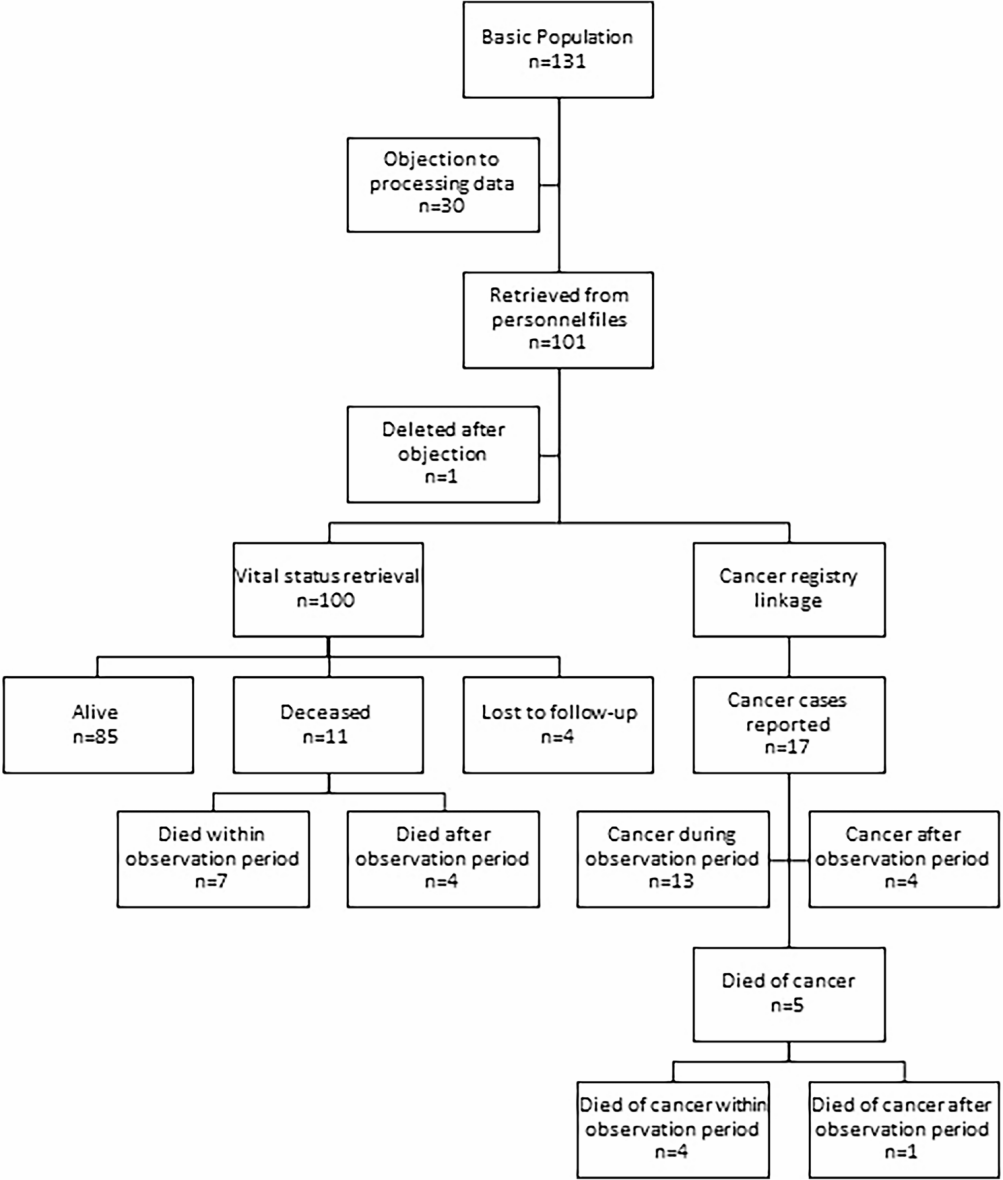
Overview of retrieved cases from personnel files, vital status retrieval and matching in the cancer registry. note cancer cases do not equal persons

The results of the retrieval of the vital status and linkage with the cancer registry are presented in Fig. 1.

Demographics for the 100 firefighters can be found in Table 1. All former and current firefighters who had worked for the Neubrandenburg municipal fire brigade for at least three months were included in the analyses. This was true for all cases.

**Table 1:** Descriptive demographics for the firefighter cohort

	*N* (Percentage)	Mean	Min	Max
Total N	100 (100%)	-	-	-
Sex (male)	99 (99%)	-	-	-
Age at end of observation period (2016)	-	54.4	30	75
Employment duration in years	-	28.7	0.5	43.8

### SIR-main-analysis

Only the cancer diagnoses confirmed within the observation period (*n* = 13) were used in the main analysis of the SIR.

During matching, an implausible low number of overall cancer cases was found in the CR-MWP from 1990 to 2001. This is further explained in the discussion. Accordingly, it was necessary to restrict the observation period for the SIR analysis to 2002–2016 as to not underestimate the number of cancer cases. The analysis of the SMR remains unaffected by this as no deaths were identified before 2002. The results of the main SIR analysis are shown in Table 2.

There are no significant differences in the incidence of all cancers (C00-97 w/o C44) compared to either the reference population of MLD or MWP. Among the specific entities, there is a significantly higher incidence of bladder cancer in firefighters compared to both reference populations (MLD: SIR 11.14, 95% CI [1.35;40.26]/MWP: SIR 9.78, 95% CI [1.18;35.33]).

**Table 2:** Results of SIR-Main- analysis for the observation period 2002–2016

		Reference population					
		Mecklenburg Lake District			Mecklenburg-Western Pomerania		
Diagnosis	Number of cases	SIR	SIR CI-	SIR CI+	SIR	SIR CI-	SIR CI+
All cancers (C00-97 w/o C44)	13	1.37^a^	0.63	2.51	1.38^b^	0.63	2.62
Digestive organs and peritoneum (gastrointestinal tract) (C15-C26)	< 5	0.59	0.02	3.31	0.64	0.02	3.56
Trachea, bronchi and lung (C33-C34)	< 5	2.24	0.27	8.09	2.13	0.26	7.71
Prostate (C61)	< 5	1.72	0.21	6.22	1.74	0.21	6.30
Testicles (C62)	< 5	8.27	0.21	46.09	7.32	0.19	40.77
Bladder (C67)	< 5	11.14	1.35	40.26	9.78	1.18	35.33

For one of the bladder cancer cases, only a D diagnosis (in situ tumour) was reported in the MWP Clinical Cancer Registry, but 13 years later the cause of death was recorded as bladder cancer. The project team decided to include this case in the incidence analysis because a transition from in situ histology to invasive carcinoma can be assumed in the long period between reporting the D diagnosis and death from bladder cancer. Excluding this case from the incidence statistics would likely underestimate the SIR. The time of diagnosis was assumed to be half way within the period between the reported D diagnosis (2000) and the time of death (2013)

*SIR* Standardized incidence ratio, *CI* Confidence interval

^a^*p* = 0.241

^b^*p* = 0.211

### SIR-sensitivity-analysis

Four cancer cases that occurred after 2016 were included in a sensitivity analysis, extending the observation period to 2019. This means a total of *n* = 17 cancer cases could be included in this analysis.

The results of this SIR analysis are shown in Table 3. There are no significant differences in the overall cancer incidence between the recorded firefighters and the reference populations. While the main analysis showed a significantly increased incidence of cancer of the bladder compared to the reference populations, the sensitivity analysis did not reach statistical significance (MLD: SIR 8.21, 95% CI [0.99;29.66]/MWP: SIR 6.89, 95% CI [0.83;24.90]).

**Table 3:** Results of SIR-Sensitivity-Analysis for the extended time period 2002–2019

		Reference population					
		Mecklenburg Lake District			Mecklenburg-Western Pomerania		
Diagnosis	Number of cases	SIR	SIR CI-	SIR CI+	SIR	SIR CI-	SIR CI+
All cancers (C00-97 w/o C44)	17	1.44	0.77	2.47	1.43	0.76	2.45
Digestive organs and peritoneum (gastrointestinal tract) (C15-C26)	< 5	0.42	0.01	2.35	0.45	0.01	2.49
Trachea, bronchi and lung (C33-C34)	< 5	1.67	0.20	6.02	1.54	0.19	5.55
Prostate (C61)	< 5	2.30	0.63	5.88	2.27	0.62	5.82
Testicles (C62)	< 5	6.82	0.17	38.01	6.25	0.16	34.82
Bladder (C67)	< 5	8.21	0.99	29.66	6.89	0.83	24.90

For one of the bladder cancer cases, only a D diagnosis (in situ tumour) was reported in the MWP Clinical Cancer Registry, but 13 years later the cause of death was recorded as bladder cancer. The project team decided to include this case in the incidence analysis because a transition from in situ histology to invasive carcinoma can be assumed in the long period between reporting the D diagnosis and death from bladder cancer. Excluding this case from the incidence statistics would likely underestimate the SIR. The time of diagnosis was assumed to be the middle of the period between the reported D diagnosis (2000) and the time of death (2013)

*SIR* Standardized incidence ratio, *CI* 95% Confidence interval

### SMR-analysis

By December 31, 2016, a total of seven people in the observation cohort had died. Cancer was coded as the cause of death for four of them (57.1%). Table 4 shows the results of the SMR. There are no significant differences between the firefighters and the two reference groups in terms of mortality from all cancers (C00-97 w/o C44). However, there are statistically significant higher values for deaths from bladder cancer compared to both the district and the federal state reference group.

**Table 4:** Results of the SMR-analysis for the observation period 1990–2016

		Reference population					
		Mecklenburg Lake District			Mecklenburg-Western Pomerania		
Cause of death	Number of cases	SMR	SMR CI-	SMR CI+	SMR	SMR CI-	SMR CI+
All cancers (C00-97 w/o C44)	< 5	1.03^a^	0.28	2.64	1.07^b^	0.29	2.75
Trachea, bronchi and lung (C33-C34)	< 5	1.04	0.03	5.79	1.02	0.03	5.66
Prostate (C61)	< 5	8.96	0.23	49.95	8.95	0.23	49.85
Bladder (C67)	< 5	29.25	3.54	105.66	29.69	3.60	107.24
All causes of death (2002–2016)	7	ND	ND	ND	1.05	0.42	2.15

*SMR* Standardized mortality ratio, *CI* Confidence interval, *ND* No data available

^a^*p* = 0.544

^b^*p* = 0.511

## Discussion

This study investigated whether the incidence and mortality of cancer among the firefighters of the Neubrandenburg municipal fire brigade differs from the respective expectation based on two reference populations. The main analyses of the SIR and SMR revealed no significant differences in incident or mortality of all cancers (C00-97 w/o C44) between the firefighters and the reference populations. However, the analyses revealed a significantly higher incidence and mortality of bladder cancer among the firefighters compared to the reference populations (MLD: SIR 11.14, 95% CI [1.35;40.26]; SMR 29.25, 95% CI [3.54;105.66]/MWP: SIR 9.78, 95% CI [1.18;35.33]; SMR 29.69, 95% CI [3.60;107.24]). This finding can be linked with results of a biomonitoring study from Germany [2]. The study examined employed and volunteer emergency personnel from fire departments as well as employees from respiratory protection and hose workshops in Berlin and Hamburg. The participants were asked to provide urine samples at three different time points after their respective tasks [27]. The analysis showed that the 1-hydroxypyrene (1-OHP) value, a metabolic product of the PAH pyrene was above the limit of quantification more often after than before deployment. Even though the PAH pyrene, whose metabolite 1-OHP was measured in the urine samples, is not itself considered carcinogenic, the measured values show that PAHs are absorbed in fire incidents [27].

The marginal statistical significance for the SIR of bladder cancer is based on our decision to include one case that was reported as a D-diagnosis and later as deceased because of bladder cancer, without a C-diagnosis being reported in between. The time of diagnosis was assumed to be the middle of the period between the reported D diagnosis and the time of death. The reference population contains all deceased cancer cases but not the D information. For cases with no prior incidence notification the year of death is used as the incidence year. Excluding this case would lead to a lower statistically non-significant SIR (MLD: 5.5; MWP:4.8). In regards to the two reference populations no notable differences in SIR and SMR could be found. This indicates that no regional factors influenced the respective rates. Therefore, both rates are equally valid.

Three further written objections were received only after data analyses had already been finished. In accordance with the GDPR (Sect. 7 [3]) these cases were not retrospectively excluded since after conduction of the analysis results contained no individual related data anymore and results consisted of aggregated tables exclusively. A total of 30 currently employed firefighters did not respond to the letter by the HR department of the city of Neubrandenburg. They were not included in the analysis. Hence, the study team only had limited data on the former and current firefighters employed by the Neubrandenburg municipal fire brigade. As a consequence, it was not possible to record the entire cohort of firefighters in this fire brigade between 1990 and 2016. Therefore, all reported results refer exclusively to the subset of firefighters whose data could be evaluated. It cannot be ruled out whether or not the missing consents are randomly distributed across the target population or rather follow a specific selection pattern, which could indicate a bias. In particular, if people who are not suffering from cancer and therefore may have less interest in the study have refused to participate in the study, the willingness to participate would tend to be higher among cancer patients. The cancer risk in the target population would then be overestimated on the basis of the subgroup who participated in this study. Alternatively, it is possible that our analysis underestimates the cancer risk of the target population, if primarily people with cancer had refused to participate in the study. In order to create one of the described distortions, it is not necessary that all of the persons did object for a systematic reason. As the total number is small, even the systematic omission of a few individuals could lead to a distortion. The only known information about the active firefighters that did not consent to their data being used is that they were still alive and working at the fire brigade at the time. The very wide confidence interval is the result of the low number of the recorded population. This is also reflected by the low statistical power of the analyses (power of SIR main analyses MLD: 9%, MWP: 9.1%; power of SMR analyses MLD: 4.7%, MWP 4.4%). This brings an uncertainty to the results as less common cancers might not have been captured in the data. Even if the missing 30 persons had consented to their data being used, the total number would still be quite small. This would probably still lead to low statistical power and wide confidence intervals. Therefore, more fire brigades should be included if a follow-up study is conducted. Preferably, this would include all major fire brigades in Germany. This would also allow for more nuanced analyses, e.g. stratified by the employment duration, which were not feasible with this small cohort. In future studies it should also be considered to include diseases besides cancer, e.g. respiratory diseases. This could help to further increase the knowledge about health risks for firefighters and increase necessary protection.

A key strength of the present study is the use of data from the spatially and temporally detailed population and mortality data of the comparison regions. The causes of death were made available to the study team by the Statistical Office of MWP and the CR-MWP. The expected values standardized for age group and gender could therefore be calculated precisely. The cohort matching with the CR-MWP represents a further strength of the study, as reliable data on cancer incidence is available for the period under consideration [28]. However, for the observation period from 1990 to 2001, the recorded case numbers of the cancer registry are implausibly low. This is probably due to the restructuring and reorganization of the cancer registry after the German reunification in 1989. In some cases, the reason for treatment was missing from the cancer registry data. This poses a problem for the calculation of the incidence, as this item relates to initial diagnoses and, for example, distinguishes incident cancers from reports of metastases and recurrences that must not be included in the incidence calculation. In cases where traceability was not possible, the date of the first plausible report was used as the incidence date. The consequence might be that a person is not recorded as having an incidence on the date of the actual initial diagnosis, but only at a later date. This could be the case, for example, if the initial diagnosis was registered in another federal state and this information was not forwarded to the CR-MWP in accordance with the regulations.

The sensitivity analysis, which considered further cancer cases up to 2019, did not reveal any significant differences. An extension beyond this was not carried out, as it had to be assumed at the time that there would be still a significant underreporting in the cancer registry for the final years 2020 and 2021 due to delayed notifications. If these calendar years had been included in the sensitivity analysis, the actual SIR value would most likely have been underestimated. Nevertheless, all notifications already available in the cancer registry were compared with the cohort of recorded employees. This did not result in a match.

The cause of death statistics for the period of observation were provided by the Statistical Office of MWP. All death certificates issued by physicians in MWP are transferred to the statistical office where the leading cause of death is abstracted by trained personnel. Diagnoses are coded according to ICD-10 [25], which allows to differentiate cancer entities. Only in very few cases the cause of death is unknown, usually requiring a police investigation. If an actual cancer was not recorded on the death certificate it would lead to an underestimation of the cancer risk among firefighters.

The results of the analyses must be discussed against the background of the existing methodological limitations. As no data on individual exposure were used, the results presented in the analyses must not be interpreted in the sense of a direct causal relationship between exposure to carcinogenic substances in the Neubrandenburg municipal fire brigade and cancer incidence and/or mortality.

In 2023, the IARC published a monograph on occupational exposure as a firefighter, concluding that there is sufficient evidence that exposure is carcinogenic and causes mesothelioma and bladder cancer. The IARC also found limited evidence for cancers of the colon, prostate and testis as well as malignant melanoma of the skin and non-Hodgkin lymphoma [29]. In conclusion the IARC determined a group 1 classification for occupational exposure as a firefighter for all categories and types of firefighters. Our findings regarding bladder cancer incidence and mortality are in line with the results published by the IARC.

It is interesting to note that for some types of cancer there is clear evidence in the literature of an increased incidence compared to the reference populations, but less evidence of increased mortality. One could speculate whether this is related to better early detection and treatment among firefighters. A large number of the studies explicitly cite the healthy worker effect [3–5, 7–10, 12, 14, 17–21, 30–33]. In epidemiological studies, the healthy worker effect generally describes a difference between occupational groups and the general population caused by the withdrawal of seriously and/or chronically ill people from the labour market. More specifically, the healthy worker effect can also be influenced by the selection of particularly healthy people for a particular job. This can be the case for physically demanding activities that are physically impossible for people with chronic illnesses and/or disabilities [34, 35].

The healthy worker effect can be avoided or at least reduced in epidemiological studies if a suitable comparison group is selected, e.g. employees with a similarly physically demanding job or a job with similar recruitment requirements but without the exposure of interest [34–36].

The research team therefore discussed possible alternative reference populations. It was considered that a cohort of police employees could be a suitable reference, as they have similar recruitment requirements, for example, but they do not face the same exposures as firefighters. Unfortunately, no corresponding data could be collected. Another option could have been to divide the sample by employment time, as shorter employment time would correlate with lower exposure. This was not possible as the overall sample was already quite small and further stratification would have led to even greater uncertainty of the results. It is very important to consider the healthy worker effect when interpreting the results, as even non-significant differences can already indicate trends in a supposedly healthier population. In this study, the risk of the investigated firefighters should be lower compared to the slightly less healthy general populations of MLD and MWP. The mortality from all causes of death of the firefighters may be slightly underestimated in the SMR analysis, but is still comparable to that of the reference population of MWP, so that no healthy worker effect was observed in our study.

## Conclusions

Overall, the results of the analyses provide evidence of a significantly increased risk of bladder cancer among the firefighters in the Neubrandenburg municipal fire brigade. However, there are non-significant trends in the data that should not be ignored. The results of the sensitivity analysis show an increase in observed cases compared to expected cases, particularly for all cancers (C00-C97 w/o C44) and prostate cancer. In addition, the SMR analysis shows a significantly increased mortality rate for bladder cancer in the surveyed firefighters compared to both reference populations.

The increased incidence and mortality of bladder cancer are in line with published results in the literature, especially the 2023 IARC monograph on occupational cancer risks among firefighters. Due to the long latency periods for cancer, it should be considered to revisit this cohort in the future to follow up on the observed trends. One aim should be to convince firefighters who did not want to be included of the importance of their consent in order to minimize potential sources of bias in the analysis.

## Data Availability

The datasets generated and/or analysed during the current study are not publicly available to protect the privacy of the study participants, but aggregated anonymized data are available from the corresponding author upon reasonable request.

## Abbreviations

1-OHP: 1-hydroxypyrene
CI: Confidence interval
CR-MWP: Cancer registry of Mecklenburg-Western Pomerania
DGUV: German Social Accident Insurance
HR: Human Resources
IARC: International Agency for Research on Cancer
IPA: Institute for Prevention and Occupational Medicine
MLD: Mecklenburg Lake District
MWP: Mecklenburg-Western Pomerania
NA: Not applicable
PAH: Polycyclic aromatic hydrocarbons
PPE: Personal protective equipment
SIR: Standardized incidence ratio
SMR: Standardized mortality ratio
TTP: Trusted Third Party
UMG: University Medicine Greifswald
